# Mental Suffering in Protracted Political Conflict: Feeling Broken or Destroyed

**DOI:** 10.1371/journal.pone.0156216

**Published:** 2016-05-27

**Authors:** Brian K. Barber, Clea A. McNeely, Eyad El Sarraj, Mahmoud Daher, Rita Giacaman, Cairo Arafat, William Barnes, Mohammed Abu Mallouh

**Affiliations:** 1 University of Tennessee, Knoxville, Tennessee, United States of America; 2 New America, Washington, DC, United States of America; 3 Gaza Community Mental Health Programme, Gaza Strip, Palestine; 4 Independent Consultant, Gaza Strip, Palestine; 5 Birzeit University, Birzeit, West Bank, Palestine; 6 Save the Children Foundation, Ramallah, West Bank, Palestine; 7 Georgetown University, Washington, DC, United States of America; 8 Al-Qattan Foundation, Gaza Strip, Palestine; Georgetown University Medical Center, UNITED STATES

## Abstract

**Purpose:**

This mixed-methods exploratory study identified and then developed and validated a quantitative measure of a new construct of mental suffering in the occupied Palestinian territory: *feeling broken or destroyed*.

**Methods:**

Group interviews were conducted in 2011 with 68 Palestinians, most aged 30–40, in the West Bank, East Jerusalem, and the Gaza Strip to discern local definitions of functioning. Interview participants articulated of a type of suffering not captured in existing mental health instruments used in regions of political conflict. In contrast to the specific difficulties measured by depression and PTSD (sleep, appetite, energy, flashbacks, avoidance, etc.), participants elaborated a more existential form of mental suffering: feeling that one’s spirit, morale and/or future was broken or destroyed, and emotional and psychological exhaustion. Participants articulated these feelings when describing the rigors of the political and economic contexts in which they live. We wrote survey items to capture these sentiments and administered these items—along with standard survey measures of mental health—to a representative sample of 1,778 32–43 year olds in the occupied Palestinian territory. The same survey questions also were administered to a representative subsample (n = 508) six months earlier, providing repeated measures of the construct.

**Results:**

Across samples and time, the *feeling broken or destroyed* scale: 1) comprised a separate factor in exploratory factor analyses, 2) had high inter-item consistency, 3) was reported by both genders and in all regions, 4) showed discriminate validity via moderate correlations with measures of feelings of depression and trauma-related stress, and 5) was more commonly experienced than either feelings of depression or trauma-related stress.

**Conclusions:**

Feeling broken or destroyed can be reliably measured and distinguished from conventional measures of mental health. Such locally grounded and contextualized measures should be identified and included in assessments of the full impact of protracted political conflict on functioning.

## Introduction

Hundreds of millions of people are affected by political conflicts [1], defined as conflicts between groups due to an uneven distribution of land, social justice, resources, or cultural or religious power [2]. Many conflicts are protracted, include political violence, and create extended suffering for millions, including mental suffering.

The vast majority of studies assessing the impact of political conflict on mental suffering focus on depression and post-traumatic stress disorder (PTSD), often using quantitative assessment instruments developed in the West on populations not experiencing political conflict. [3–6]. There are vigorous arguments among scholars as to the appropriateness of using standardized, Western measures of depression and PTSD across cultures (e.g., [6–9]). However, regardless of one’s position on the universality of these constructs, it is valuable to also seek locally-defined (i.e., emic) measures of mental suffering, particularly if such measures differ conceptually and empirically from standard Western measures. Knowing precisely how a population suffers can guide practices and policies to minimize suffering. For example, if mental suffering is found to be inextricably linked to social and political injustice, then amelioration of that suffering may require social justice and political solutions in addition to the individualized approaches commonly used to reduce suffering from depression and PTSD [10–13].

In this paper, we describe the identification and then the development and validation of a quantitative measure of a new construct of mental suffering (see [14] for a published abstract) in a region of one of the world’s most protracted political conflicts, the occupied Palestinian territory (oPt). Palestinians are an apt population for a study of local conceptualizations of mental suffering because they have lived under political occupation and its consequences of economic and physical hardship and denial of civil liberties for at least three generations. The cohort studied here, those in their 30s, are the storied generation of stone throwers of the first Palestinian intifada (1987–1993), when as many as 90 percent participated in political activism against the Israeli occupation [15]. Since then, they have experienced a steady decline in economic and political conditions in the oPt [16, 17]. Examples of their prolonged stress include widespread political imprisonment over their life course (26 percent of all men [18]); growing mobility restrictions, including a complete blockade on the Gaza Strip since 2007 [19]; increasing unemployment and resource inadequacy [20, 21]; and limited and declining access to adequate healthcare, potable water, and basic sanitation [21–23].

A small but growing literature exists on locally-defined measures of mental suffering in politically conflicted societies, including *distress* (Afghanistan; [24]); *political violence-related stress* (Peru; [5]); *main and most distressing problems* (Afghanistan; [8, 25]); *functional impairment* (Indonesia; [26]); *spirit possession* (Northern Uganda [27, 28]); and *problems* stemming from ethnic cleansing (Rwanda; [29]). (See [30] for a review of cultural concepts of distress in trauma situations). The fact that locally-defined measures of mental suffering have been found in every setting of political conflict explored by researchers indicates the importance of elucidating these constructs and understanding to what extent they overlap (or not) with Western constructs employed by relief agencies and personnel trained in Western psychological paradigms.

Our initial purpose in undertaking the study was to elucidate the broader construct of functioning among Palestinians of this cohort, rather than mental suffering specifically. We use the term functioning as it is used by the World Health Organization [31] to encompass activities, participation, tasks, actions, body functions, etc. This broad concept extends beyond disability, impairment, or health and instead incorporates a holistic view of quality of life. By using an inductive approach that did not target the assessment of mental health or mental suffering, but rather tried to understand how a cohort of Palestinian adults conceives of functioning generally, we were able to “situate and contextualize local conceptions of suffering and wellbeing amidst the terrain of broader functioning that participants themselves chart.” (p. 91) [32]. In other words, this study provides insight into the source of mental suffering and not just its nature. In addition, we explored gender differences in mental suffering. Gender structures Palestinians’ roles in every arena of life, from family life to economic life to political life. Gender also structures exposure to political conflict and the nature of participation in political activism. Finally, we report on the reliability of measurement over time of the newly-identified construct of mental suffering and compare it to measures of trauma-related stress and feelings of depression to determine the extent to which it overlaps with Western conceptualizations of mental distress.

Numerous local Palestinian experts who are professionals in public health, psychology, political science, and psychiatry (including five authors on this paper) were consulted throughout the project. Palestinian experts provided in-depth knowledge and experience of the political conflict, insight into Palestinian culture, guidance in use of language, and suggestions regarding how best to conduct research in the oPt.

## Materials and Methods

We describe the two phases of this mixed methods study separately, as the first interview phase was completed prior to and informed the methods of the second phase. Both phases of the project were approved by the Institutional Review Boards of the University of Tennessee, Knoxville and the Palestinian Center for Policy and Survey Research (PSR) in Ramallah, West Bank, whose trained field workers collected all of the data. Participants provided written consent to participate in the study.

### Interview Phase

#### Sample

In February 2010, trained fieldworkers from PSR interviewed 68 adults (33 men, mean age 34.8 years, range 21–53; 35 women, mean age 32.2 years, range 20–49) in the West Bank, East Jerusalem, and the Gaza Strip in fourteen groups of 5 same-sex individuals each. (Two Jerusalem men were absent from their interview because of transportation restrictions, resulting in a total sample of 68). PSR fieldwork supervisors purposively selected participants to assure representation of gender, region, refugee status, and (in the Gaza Strip) the two main political factions of Fatah and Hamas. The majority of participants were married (71% of women; 85% of men) and approximately half were employed (55% of men [45% of men in Gaza]; 44% of women). The group interviews were conducted in rented rooms in office buildings.

Each group interview lasted 60–90 minutes and was conducted in Arabic and audio recorded by PSR fieldwork supervisors. The first author (male) and a female U.S. graduate student attended the group interviews. They sat on the periphery with a translator, who did simultaneous translation so they could monitor the progress of the group interview and provide guidance to the interviewer as necessary. The participants were explained the purpose of the research (to understand what quality of life meant to Palestinians of their cohort) and were given a brief description of the first author’s extensive experience in the oPt and previous research of Palestinians of their age cohort. The participants were asked three questions in Arabic to assess how they conceived of functioning or well-being. First, they were asked: ‘Think of two people who you know well; one who you think is doing relatively well in life and the other who you think is not doing well in life. Please describe both of these people.’ Participants were asked to use a pseudonym and to reveal no identifying information about the individuals they described. Subsequently, the participants were asked to list the main domains of functioning and then to prioritize that list to the three most important domains.

PSR staff transcribed the audiotapes and translated them into English. To assure proper translation and interpretation, the original Arabic transcripts of all sections of text related to the new construct of mental suffering were translated independently by the seventh author of this paper, who had not seen the original translation. This validation work was critical because, as described below, we used the actual language of research participants to name the construct and to quantitatively measure it.

#### Analyses

The focus of the analysis was to identify distinct domains of functioning and craft survey items that could be used for a population-based survey of functioning. Content analyses of the English transcripts were done with Atlas.ti (version 6.2.27) individually and jointly by the first, third, and fifth authors, all from the United States. Their experience with Palestinian culture ranged from long-term residence in Palestine to no experience. Coding of the interview transcripts proceeded systematically, following a grounded theory approach, including independently coding selected sections or full interview transcripts, multiple interpretive sessions [33], and repeatedly checking and validating with the local experts (see [32] for a full description of the coding process).

First, immediately after the group interviews, the first author and a graduate assistant, who had sat in during the interviews, established a provisional list of main domains of functioning that were apparent in their recollections and notes of the interviews. Next, three coders, including the first and third author, independently open coded sections from 8 of the 14 group interviews to confirm or adjust the preliminary list of domains. All coders received intensive training in Atlas.ti and in qualitative data coding. In a series of interpretive sessions [33], this team of three coders finalized the set of main domains to include economic, education, employment, family, health (mental and physical), personal characteristics, political and religious functioning. The coding team then open-coded a subset of the interviews to develop sub-codes for each domain and, in further interpretive sessions, refined the sub-codes to between 1 and 22 sub-codes per domain. The full set of interviews was then coded using this coding structure, with each member of the coding team coding separate interviews.

The coding structure was shared regularly throughout the coding process with eight local Palestinian experts (including the second, fourth, fifth, sixth and last author) in face-to-face meetings in the oPt and through email. To assess consistency of the application of the coding scheme coding across coders, a single interview was coded three times by three different coders, all using the same coding scheme developed through the iterative process described above. We then compared the three versions of the coded interview to assess consistency between coding schemes. The large majority (73%) of the quotations that were coded by any one coder were also coded by all three coders. When a difference did exist among the coders, it most often (68%) occurred when two raters had coded a quotation similarly but the third rater failed to code that specific quotation. It was rare (2%) that any quotation was coded differently by two or more coders.

### Survey Phase

The results of the coding revealed the central role of the political domain of functioning—ranging broadly from the occupation as a political system to personal perceptions of political identity and solidarity; see [32] for details. The political domain of functioning was seen as impacting all other domains of functioning (e.g., economic, education, family, psychological). Relative to references to psychological functioning, the large majority of references invoked the political conflict as the source of psychological suffering. It was in those sections of the narratives that we noted the unique focus on an existential form of psychological suffering distinct from the somatic symptoms and feelings that comprise common constructs of emotional distress such as depression, anxiety, grief, and post-traumatic stress. We created or identified existing survey items to assess mental suffering along with multiple other domains of functioning. We consulted on the drafting of these items with key advisors as well as enlisting them to assist in reducing the final item set. The survey was translated into Arabic by PSR staff and back translated into English by other bilingual Palestinians. (See [34] for details of this phase of the project).

#### Study Design and Samples

A retrospective cohort study was conducted. PSR used a three-stage probability sampling strategy (individuals within households within population clusters) to achieve a representative sample of 1,778 30–40 year old Palestinians in the West Bank, East Jerusalem, and the Gaza Strip drawing from all Palestinian Central Bureau of Statistics enumeration areas from updated 2007 census maps. Kish tables were used to select one eligible adult per household. Access to a representative sample was made possible because PSR maintains up-to-date census maps and a staff of trained field workers and supervisors in Gaza, East Jerusalem and the West Bank to conduct regular opinion polls and provide nonpartisan research and analysis. Trainings for field supervisors and interviewers were conducted separately in the West Bank/Jerusalem and the Gaza Strip due to travel bans between the regions.

Prior to administering the survey to the main sample of 1,778, we pilot tested the measures in June 2011 with a survey administered to a representative sub-sample of 508. We conducted principal components analysis on the items measuring mental suffering in the pilot survey to determine if we could reduce the number of items used to measure each construct. A shortened version of the pilot survey was then administered to the full sample of 1,778 in October/November, 2011. Both surveys were administered via household interview, with pairs of interviewers (a man and a woman) visiting each sampled home. The pilot survey took under an hour to complete, and the full survey took 60–90 minutes. The full survey contained an extensive event history calendar used to document events over the past 25 years (1987–2011), although data from the event history calendar are not used in the current data analysis. Response rates for both the pilot survey and the survey of the main sample exceeded 90%.

We analyzed four datasets from these two samples. First, the pilot sample of 508 (pilot sample) was also re-interviewed as part of the main study sample, providing data at two time points. We refer to data for these 508 respondents from the first time point as the pilot data and the data from the second time point as the replication data, respectively. Second, the main sample consists of 1,778 respondents, which includes the 508 individuals from the replication dataset plus an extended sample of 1,270, which is also representative.

The main sample of 1,778 had high levels of exposure to political violence. Between 1987 and 2011, 78% had their home raided by Israeli military forces, 74% had witnessed others being humiliated by Israeli or Palestinian military or police and 62% had been verbally abused themselves. Over half (56%) had been shot at and 43% had been hit or kicked by soldiers or police, and 26% of men have been politically imprisoned. Forty-four percent of respondents had material loss of homes or land due to confiscation or destruction by the Israeli government. Palestinians in this representative sample also experienced a high prevalence of movement restrictions, with 68% of respondents in the West Bank reporting not being able to access medical care at some point between 1987 and 2011 due to checkpoints, barrier walls, or curfews. Approximately 1 in 5 participants (21%) rated the Palestinian government as stable or very stable, and just 27% reported high levels of freedom to express their opinions outside of the home.

#### Measures

The measure of the new construct identified in the qualitative phase of the study—feeling broken or destroyed—is described at the end of the qualitative results section. We compare that measure with existing measures of mental suffering. *Feelings of depression* consists of five items of the 8-item Patient Health Questionnaire (PHQ-8; α = .83 in the full sample; [35]). Participants were asked how often in the past two weeks they had been bothered by: trouble falling asleep, feeling tired or having little energy, poor appetite or overeating, trouble concentrating, and moving or speaking slowly/being fidgety or restless. Response options were “not at all” (0) to *“nearly every day”* (3). The number of items was reduced from eight to five due to space constraints in the survey. The five items were chosen because they had the highest factor loadings in a principal components analysis of all measures of mental suffering in the pilot sample. In the pilot sample, the correlation between this reduced set of items and the full PHQ-8 was .95. [34].

*Trauma-related stress* was measured with 5 items of the 17-item PTSD Symptom Scale (PSS) checklist (α = .90 in the full sample; [36]). As with feelings of depression, the number of items in the PSS checklist was reduced through principal components analysis using oblique rotation to accommodate space constraints in the survey of the main sample. To adapt the measure to the context under study, participants were first asked to think about harsh events they experienced during the political conflict over the course of their lives. Then, with reference to the past two weeks, they were asked to indicate whether they had experienced: recurrent or intrusive thoughts; sudden reliving of the event(s); being emotionally upset when reminded of the event(s); persistently made efforts to avoid thought or feelings associated with the event(s); and persistently made efforts to avoid activities, situations, or places that reminded them of the event(s). Responses ranged from “not at all” (0) to *“nearly every day”* (3). In the representative pilot survey, the correlation between the reduced set of items and the full 17-item PSS was .87. [34]. Very few cases were missing data on these items. If respondents were missing one or two items on either measure—trauma-related stress or depressive symptoms—the mean of the remaining items for the individual was used to assess the construct. No respondents were missing more than two items on each measure, resulting in complete data for the entire sample.

#### Analysis

For each of the three measures of mental suffering, we calculated Cronbach’s alpha and descriptive statistics (means and standard deviations) and, using the main sample, compared the levels of suffering between men and women and between the three regions of the occupied Palestinian territory using independent t-tests and one-way analysis of variance, respectively. We adjusted the p-values of the significance tests for the comparisons of mental health measures across regions using the Scheffé method. Next, in three datasets—the pilot data, the replication data, and the extended data—we correlated the measures of the newly-identified construct of mental suffering with the measures of trauma-related stress and feelings of depression to assess discriminant validity. Correlations were conducted within gender and region subgroups for each sample. All analyses were conducted with Stata 14.1 using sampling weights and the suite of svy commands to accurately estimate standard errors.

## Results

### Interview Phase

The construct for broken/destroyed was identified upon close examination of the sub-codes for the political and health domains. It was the actual Arabic words for broken/destroyed, or synonyms of them, that lead us to identify pieces of narrative describing this condition. Specifically, participants articulated (in Arabic) characterizations of suffering, such as being or feeling: broken, crushed (محطمة muḥaṭṭima), shaken up (مهزوزة mahzūza), destroyed, (مدمرة mudammira), and exhausted, tired (تعبانة ta'bāna). The aspects of the sufferer were described as the self or spirit (النفس an-nafs), morale (المعنويات al-ma'naūiyyat), and hopes or ambitions for the future (أمالك أو طموحك بالنسبة للمستقبلamālak aū ṭamūḥatak ban-nisba lil-mustaqbil).

To illustrate, following are some excerpts from the narratives that we coded. As his exemplar of a man not doing well in life, one West Bank man chose to describe a man who had spent years as a political prisoner. The man emerged from prison with “many psychological problems” which the participant described as surrounding economic difficulties, inability to complete his education, being refused to travel for not having the proper ID, and failure to marry. “His psyche/soul was very broken.” Similarly, a Gazan woman said “there are lots of people whose psyches were destroyed because the person was [psychologically] shaken. The person might not be able to bear it; the war and the problems affect them.”

A Gazan woman articulated the exhaustion and futility of achieving an education and attributed it to the political context: “For example, in our country, when you finish your education, you will exhaust yourself for many years, and will not get what you aspire for; you finish your education, and hang the certificate on a wall in your house, and won’t find any job and become worthless. We have doctors, Engineers, and accountants; it’s possible that any unskilled worker might become more important and more valuable than them. All of this is a direct effect of the occupation we live under.”

Again invoking education, a West Bank man blamed the first intifada for a collapse of the educational system in Palestine: “Education was for those who had money or the ability to study. Everyone else who needed money for other things, didn't study. They didn't finish university. This started to demolish the cornerstone and style of education. The educational system was changed. It broke the morale of the Palestinians, and even today, we suffer from this.”

A Gazan man linked feeling broken to the political and economic pressures in the Gaza Strip: “The siege must be ended because it is the source of most problems and disasters. Be it lack of income and living in need. Be it a patient who cannot travel abroad (for treatment) and dies at the crossing, or dies here or there. All of this affects the personal life of people. For example, when I come home and my son asks for a shekel and I do not have it, my spirit gets broken.”

The same sentiment was articulated by a West Bank man who, after discussing the inability to pursue education or to build homes because of the (first) intifada, said “The situation destroyed our morale… It broke our morale and character and we still suffer from this until now.” Another Gazan man said that the occupation has “affected the people psychologically in terms of killing the ambition and aspirations of the people.”

A West Bank woman explained that the “broken” house/family she had used to illustrate poor functioning was due to the father’s imprisonment. “I saw them myself, they looked like broken spirits, as if they have emotional and psychological problems, all of them, him and his sisters and brothers. All of them were suffering emotional and psychological illness.”

A Gazan man referred to the political conditions that “pile up”: “There are people in prison; people whose futures have disappeared. There are people that have physical disabilities that have destroyed their ambitions for the future.” A woman from East Jerusalem said that her exemplar of someone not functioning well was “broken psychologically” after he received a fine for building without a permit.

Our next step was to render the construct in the form of survey questions to be used in the quantitative phases of the project so that we could assess how broadly such feelings are experienced across the population and to determine if the item set would demonstrate reliability (i.e., inter-item consistency) and validity (i.e., discriminated from traditional measures). We wrote numerous items for several dimensions of functioning. For psychological functioning, three items presented below were retained that explicitly capture the sentiments we had coded for *feeling broken or destroyed*.

We found these sentiments unique enough to warrant subsequent quantitative testing. Thus, in close consultation with local experts, we proceeded to render the coded excerpts in quantitative survey format and tested their reliability and validity vis-à-vis two conventional measures of mental health (feelings of depression and trauma-related stress). Specifically, it was important to assess the degree to which the sentiments that were expressed in the interviews were common across the whole population, if the drafted quantitative items had adequate inter-item consistency, and if the new scale was sufficiently distinct from traditional measures of mental health to be considered an independent construct.

We wrote three items to capture the sentiments expressed in the interview narratives relative to *feeling broken or destroyed* (included are the Arabic versions that the participants responded to, as well as the transliterated versions). With reference to the past two weeks, participants were asked: “How often have you:

1. *felt that your spirit or morale is broken or destroyed*?”
  كم مرة أحسست بأن نفسيتك ومعنوياتك محطمة؟
  (Kam marra aḥsast ba'n nasfiyyatak wa ma'naūiyyatak muḥaṭṭama?)
2. *felt that your ambitions and hopes for the future are destroyed*?”
  كم مرة أحسست بأن امالك أو طموحك بالنسبة للمستقبل محطمة؟
  (Kam marra aḥsast ba'n amālak aū ṭamūḥatak ban-nisba lil-mustaqbil muḥaṭṭama?)
3. *felt emotionally or psychologically exhausted*?”
  كم مرة أحسست بأنك مرهق عاطفيا أو نفسيا؟
  (Kam marra aḥsast ba'nak murhaq 'āṭifiyyan aū nafsiyyan?)

Response options were “never” (1), “rarely” (2), “sometimes” (3), “often” (4) and “regularly” (5).

### Survey Phase

The means and standard deviations for the three measures of psychological suffering are presented in Table 1. The overall mean for feeling broken and destroyed was 3.08 (range 1–5; sd = 1.14), with levels higher in Gaza and the West Bank than in East Jerusalem, and with women reporting higher levels than men. The mean for feelings of depression was 1.08 (range 0–3; sd = 0.77), again with reported rates higher in Gaza and the West Bank than in East Jerusalem, and by women compared to men. The mean for trauma-related stress was 1.13 (range 0–3; sd = 0.93), with higher reported rates in the West Bank compared to Jerusalem and no gender differences.

**Table 1:** Range, means, and standard deviations for mental suffering measures, by region and gender, main sample.

		Total	Gaza	E. Jerusalem	West Bank	Males	Females
Construct of mental suffering	Range	Mean (s.d.)	Mean (s.d.)	Mean (s.d.)	Mean (s.d.)	Mean (s.d.)	Mean (s.d.)
Feeling broken or destroyed	1–5	3.08 (1.14)	3.12 (0.98)*	2.84 (1.22)^a^,^b^	3.10 (1.23)^b^	2.98 (1.16)**	3.19 (1.11)
Feelings of depression	0–3	1.08 (0.77)	1.04 (0.67)**	0.84 (0.71)^b^	1.14 (0.84)^b^	1.00 (0.76)***	1.15 (0.77)
Trauma-related stress	0–3	1.13 (0.93)	1.04 (0.78)	0.91 (0.89)	1.23 (1.02)	1.13 (0.91)	1.12 (0.94)
Unweighted sample size		1,778	616	167	994	884	894

*p < .05;

**p < .01;

***p <.001 (based on F-tests of overall difference between groups).

^a^ = Gaza vs. East Jerusalem, p <.05;

^b^ = West Bank vs. East Jerusalem, p < .05;

^c^ = Gaza vs. West Bank, p < .05, adjusted for multiple comparisons using the Scheffé test.

Note: All sample sizes are unweighted and means and standard deviations are weighted.

As is typical of many measures of mental health, the means for feelings of depression and trauma-related stress were skewed to the low end. In contrast, the mean for feeling broken or destroyed was at the midpoint of the scale, suggesting that these feelings were more widespread across the population. This is confirmed by examining the frequency distributions of the three scales. They are presented in Table 2. Overall, only 10% of the sample reported never experiencing feeling broken or destroyed over the preceding two weeks, compared to 24% of the sample who reported not at all having feelings of depression (p < .001) and 32% who reported not at all having feelings of trauma-related stress (p < .001) during those preceding weeks. This disparity was greatest for Gazans and for women. Just 6% of Gazans and 8% of women reported never feeling broken or destroyed over the preceding two weeks.

**Table 2:** Frequency distributions (presented as percentages) for mental suffering variables, main sample.

Construct of Mental Suffering	Total	Gaza	E. Jerusalem	West Bank	Males	Females
Feeling broken or destroyed						
Never	10.1	5.8	17.4	11.8	12.5	7.7
Rarely	20.9	21.9	24.6	19.6	22.4	19.6
Sometimes	32.2	37.8	29.3	28.8	31.4	33.0
Often	24.1	23.9	16.8	25.6	22.1	26.1
Regularly	12.7	10.6	12.0	14.3	11.8	13.6
Feelings of depression						
Not at all	24.4	23.2	33.5	23.7	28.6	20.3
Several days	49.5	53.4	48.5	46.9	48.1	50.9
More than half the days	20.0	19.2	15.6	21.4	18.5	21.6
Nearly every day	6.0	4.2	2.4	8.0	4.9	7.2
Trauma-related stress						
Not at all	32.4	30.7	43.1	31.7	31.7	33.2
Several days	28.9	38.0	25.8	22.9	29.9	27.9
More than half the days	30.7	27.2	25.8	34.1	30.5	31.0
Nearly every day	8.0	4.0	5.4	11.2	8.0	8.0
Sample Size	1,778	617	167	994	884	894

Note: All sample sizes are unweighted and percentages are weighted.

Next we calculated the inter-item consistency (Cronbach’s alpha) of the three scales for all region and gender subgroups in the pilot dataset, the replication dataset, and the extended dataset to assess the consistency of the average inter-item correlations across time within the same sample as well as across two representative samples at the same point in time. These results are presented in Table 3. For the three items comprising feeling broken or destroyed, the alphas ranged from .80 to .92 across the groups and data sets. For the five items comprising feelings of depression, the alphas ranged from .72 to .86, and for the five items comprising trauma-related stress the alphas ranged from .81 to .93.

**Table 3:** Inter-item consistency (Cronbach’s alpha) for psychological functioning scales, by gender and region, within three datasets.

Dataset	Construct of mental suffering	Total	Gaza	E. Jerusalem	West Bank	Males	Females
Pilot (n = 508)	Broken/destroyed	.82	.80	.88	.82	.80	.84
	Depressive feelings	.76	.77	.74	.75	.79	.72
	Traumatic stress	.86	.81	.88	.88	.87	.85
Replication (n = 508)	Broken/destroyed	.85	.89	.89	.83	.84	.86
	Depressive feelings	.82	.80	.79	.83	.83	.81
	Traumatic stress	.92	.92	.88	.92	.91	.93
Extended (n = 1,270)	Broken/destroyed	.84	.86	.92	.81	.84	.84
	Depressive feelings	.84	.84	.86	.83	.83	.84
	Traumatic stress	.90	.91	.92	.90	.89	.92

Note: All sample sizes are unweighted.

Finally, to test for discriminant validity, we estimated correlations between each of the three scales for all region and gender subgroups within the three datasets. Results are presented in Table 4. The correlations between feeling broken or destroyed and feelings of depression showed good discriminant validity, with correlations across subgroups and datasets ranging from .35 (p < .05) to .59 (p < .001). Similarly, the results showed good discriminant validity between broken or destroyed and trauma-related stress, with correlations ranging from .11 (n.s.) to .38 (p < .01) (Table 4).

**Table 4:** Bivariate correlations among mental suffering scales, by gender and region within three datasets.

Dataset		Total	Gaza	E. Jerusalem	West Bank	Males	Females
Pilot (n = 508)	Broken/destroyed—Depressive feelings	.46***	.50***	.35*	.46***	.44***	.50***
	Broken/destroyed—Traumatic stress	.21***	.11	.37*	.23***	.20**	.23***
	Depressive feelings—Traumatic stress	.24***	.16*	.42**	.24***	.28***	.18**
	Sample size	508	176	48	284	254	254
Replication (n = 508)	Broken/destroyed—Depressive feelings	.55***	.50***	.50***	.58***	.55***	.54***
	Broken/destroyed—Traumatic stress	.20***	.18*	.38**	.19**	.22***	.18**
	Depressive feelings—Traumatic stress	.29***	.22**	.42**	.27***	.28***	.29***
	Sample size	508	176	48	284	254	254
Extended (n = 1,270)	Broken/destroyed—Depressive feelings	.55***	.50***	.54***	.58***	.51***	.59***
	Broken/destroyed—Traumatic stress	.21***	.24***	.12	.22***	.19***	.23***
	Depressive feelings—Traumatic stress	.27***	.20***	.16	.31***	.30***	.24***
	Sample size	1270	441	120	709	630	640

*p < .05;

**p < .01;

***p < .001.

## Discussion

In a broad effort to have a unique cohort of Palestinian adults describe overall functioning in their society, we detected specific characterizations of mental suffering that are not commonly assessed in populations experiencing political conflict. In contrast to the discrete symptoms that comprise conventional measures of depression (problems with sleep, appetite, energy etc.) and trauma-related stress (recurrent or intrusive thoughts, efforts to avoid thought, places or activities, etc.), participants described a more existential form of social suffering that, according to them, was a function of the chronic and burdensome political and economic contexts that plague them. Specifically, expressions included one’s self, spirit or future being broken or destroyed and feeling psychologically and emotionally exhausted.

After writing survey items to capture these sentiments, we proceeded to test the reliability and validity of these sentiments on multiple representative samples of the same age group, in all cases attending to the gender and regional diversity of the samples. These empirical tests provided clear evidence for the viability of the new construct. In terms of reliability, the three-item *feeling broken or destroyed* scale had strong inter-item consistency across time for the same sample, across multiple samples and across subgroups within those samples. This suggests that feeling broken or destroyed is an internally consistent construct of mental suffering for both genders and in all regions of the occupied Palestinian territory.

In terms of validity, the *feeling broken and destroyed* scale distinguished itself from two conventional measures—*feelings of depression* and *trauma-related stress*—by low to moderate correlations. The independence of the construct was also evident in its distribution across the sample. The findings showed that *feeling broken or destroyed* was a more normative (i.e., commonly experienced) form of suffering among both men and women in this population, although feelings of depression and trauma-related stress also were evident.

*Feeling broken or destroyed* also appears to be distinct from other locally defined constructs of mental suffering. In other exploratory research of locally-defined constructs of mental suffering during political conflict in Afghanistan [24], Sri Lanka [37], Afghanistan [24, 25] and Darfur [38], no clear equivalent to feeling broken or destroyed was identified. However, in Peru, Quechua residents who had suffered political violence for over a decade at the hand of the Shining Path guerillas reported “being like *un pedazo de tela vieja o gastada* (an old piece of cloth, frail and worn out), something which is beyond repair and turns to powder between your fingers” (p. 210) [5]. Although this feeling was not further explored systematically in the research, there may be parallels with *feeling broken or destroyed*.

Other evidence suggests that *feeling broken or destroyed* may not be a uniquely Palestinian reaction to long-term political and economic oppression and conflict. The three-item measure was included in a national study of Egyptian youth conducted in 2014 and found to have good psychometric properties: Cronbach’s alpha = .87; mean = 2.29, sd = 1.04. [39].

### Conceptualization

Apart from empirically testing the distinctiveness of the new construct from conventional measures, we spent considerable effort discerning if and how the construct overlapped with other conceptual characterizations of mental suffering before deciding, in the end, to label it *feeling broken or destroyed*. After reviewing carefully several related constructs (see below), it became clear that no existing construct was an adequate label for the sentiments described by our participants. We decided therefore, in consultation with our key Palestinian advisors, to preserve the labeling the participants used themselves. Naturally, since this was their way of referring to the feelings and because they linked these feelings to the contexts they have lived under, this conceptual label directly reflects the political history of the conflict with Israeli as Palestinians have experienced it—that is, it is that conflict, as well as the more recent factional infighting, that has led them to feel broken, destroyed, and psychologically exhausted (see [40] for a comprehensive overview of the conflict).

Although initially peaceful, relations between the Arab residents of Palestine and waves of in-migrating Jews from Russia and Eastern Europe at the turn of the 20th Century devolved into decades of conflict over land control and nationalism for both peoples. Great Britain was unsuccessful in its mandate to govern the region after World War I, resigning that authority in 1947. By 1948, Jewish forces, with significant Western backing, defeated Arab forces and the State of Israel was created on approximately three-quarters of the region that was at that time known as Palestine. The large majority of Arabs fled and/or were forced to relocate in Jordan, Lebanon, Syria, Egypt, and beyond.

Through another military victory in 1967, Israeli forces took control of several additional territories, including the Gaza Strip (from Egypt), and the West Bank and Eastern Jerusalem (from Jordan). Ongoing tensions erupted in 1987 in the three territories (currently referred to as the oPt) via the first Palestinian intifada (Arabic for “shaking off”). This popular movement culminated in formal efforts to facilitate self-determination for Palestinians in those territories (via the 1993 and 1995 Oslo Declaration of Principles). These efforts failed to improve conditions for Palestinians, and the second intifada broke out in 2000 and lasted until 2005. This was followed in 2007 by the civil war between the Fatah and Hamas political factions (see [41] for a recent overview of Gaza) and by three wars between Israeli forces and Gazan (primarily Hamas) forces (2008–9, 2012, 2014) that have resulted in the deaths of more than 3500 Gazans and up to 100 Israelis. Economic and political conditions have worsened since 2005, particularly for Gazans, who since 2007 have been under a crippling economic blockade [42]. In short, it is easy to see from this history of persistent and worsening conflict and decline in living condition that Palestinians might often feel broken or destroyed. We close the discussion now with a brief review of related constructs, clarifying why we judged them inadequate to characterize the form of suffering we identified in this study.

*Despair* is a construct that has been invoked regularly to describe Palestinian experience. Most often, however, the construct has been used rather loosely, without much if any definition; and it does not appear to have been measured [43–47]. This lack of empirical foundation aside, our decision not to adopt despair as the label for the new construct was driven by conceptual concerns of our key advisors. For some of them, despair implied a lack of hope. Such would be unacceptable because, paradoxically, despite high levels of suffering (sometimes referred to as despair), hope—often paired with resistance—figures prominently in the master Palestinian narrative [46, 48, 49].

As our late key informant and co-author Gazan psychiatrist Eyad El Sarraj once put it: “When we hear the blasts of their bombs, we do not falter. We resist even in our silence. We hope still that one day they will send a message of justice and peace. We will always be there.” [50]. Muna, one West Bank woman from our study, elaborated the suffering and its complexity as follows: “a kind of depression or despair, and some sort of emotional or psychological disorders, I don’t want to call them physiological disease. We live a very difficult life, in our practical and educational lives, we lived a short period of those trouble and suffered a great deal, how would you imagine someone lived all along those conflicts and intifadas… suffering a great deal, and feel[ing] emotionally exhausted and psychologically ill, and having great psychological problems. If not all the Palestinians, I’m sure more than three quarters feel this way, we lost hope, but [we are] still immovable and resisting.”

Despite including characterizations of brokenness and destroyed, it is just this persisting resolve and resistance that makes the construct *mental defeat* inappropriate as a construct label; it being defined as a “state of giving up in one’s own mind all efforts to retain one’s identity as a human being and a will of one’s own.” [51]. Otherwise, the construct *brokenness* is inadequate given its association primarily with an individual’s disconnection or disruption from others, accompanied by self-blame for feeling that something is “missing” or “wrong” or one is “no longer whole.” [52]. *Demoralization* is also used occasionally in describing Palestinian experience (e.g., [53]), but the scientific literature appears thin and highly clinical. [54].

Psychological or *emotional exhaustion* was raised often enough in the narratives for us to have written a survey item to reflect it (see Muna’s comment above for one exemplar). We chose not to adopt it as the overarching conceptual label, however, because it does not cover the sense of brokenness and because the extensive literature on it implies “burnout” from overextending, overcommitting, or overworking oneself in specific “people-oriented” environments (e.g., the workplace; [55, 56]).

In short, we found no existing construct that could adequately serve as an overarching concept for the sentiments that were articulated in the narratives and that we subsequently coded, quantified, and tested. We felt most comfortable, therefore, labeling the construct as it was articulated by the participants themselves.

Retaining original language in labeling the construct assists also in underscoring the role of context is defining this form of mental suffering. As elaborated in the full analysis of the interview narratives that grounded this project [32]—and elsewhere for Palestinians [57–60] and other beleaguered peoples [5, 8, 27]—it was the political and associated economic constraints that were responsible for the sense of brokenness or destruction (see, e.g., the construct of social suffering from medical anthropology [61] and the structural violence that causes it [62]).

The political source of this form of suffering has implications for its remedy. The underlying conditions causing the feelings of being broken or destroyed will not change unless there are political changes to correct on-going violations of basic rights, economic and political self-determination, and dignity [32, 58, 59–62].

## Limitations

Strengths of this study include the careful qualitative identification of *feeling broken or destroyed* using methods that did not specifically elicit reports of mental suffering; the intensive involvement of local Palestinian experts in identifying, naming, and interpreting the new construct; and the ability to measure and test the validity of the construct in multiple representative samples. There are, of course, limitations as well. First, the analyses in the qualitative component of the study were conducted on English translations of the Arabic transcripts. Although we used multiple, independent translations of the Arabic text, we may have missed important nuances in the experiences of the Palestinian respondents. Second, the measures of feelings of depression and trauma-related stress were shortened versions of the widely validated measures of these constructs, although fortunately the correlations between the shortened and full versions of these measures in the pilot sample were high. Third, when creating the *feeling broken or destroyed* scale we used a 5-point response scale (“never” (1) to “regularly” (5)), compared to the 4-point scale (“not at all” (0) to “nearly every day” (3)) used by the developers of the feelings of depression and trauma-related stress measures. We did this in order to be more consistent with other measures of wellbeing that were developed within our larger project. Our intent in validating the new scale in this paper has not been to empirically compare average levels of these forms of mental suffering, and, of course, doing so would not have been appropriate given the differing response options across the scales. That said, any future research that would investigate mean level differences of these three types of mental suffering within any population should standardize the response options prior to new data collection. Finally, this initial identification and validation of feeling broken and destroyed occurred in a single setting and thus we cannot offer a recommendation regarding on its relevance to other conflict-affected populations.

## Moving Forward

There are at least two ways in which research should proceed in continuing the validation of the findings presented here: demonstrating relevance to other populations and specifying the unique nature of the construct.

As to the former, given that participants in this study defined the source of feeling broken or destroyed in the constraining, rights-infringing political and economic context, the construct should not be salient only to Palestinians. While the Palestinian experience may well consist of an abundance of obvious markers of demeaning political control, many other populations are oppressed or discriminated against. Important validation of the new construct, therefore, would come from assessing it in varied populations experiencing political conflict.

As to the second extension, it is one thing to demonstrate the discriminant validity of the new construct via correlations with conventional measures as we did here. It would be yet more valuable to elaborate and quantify the correlates or predictors of this type of mental suffering. To that end, work out of our project is demonstrating that feeling broken or destroyed is significantly related to health, human insecurity, unemployment, and patterns of persistent humiliation and imprisonment. [18, 32, 63, 64].

## References

[1] Harbom L, Wallensteen P. Armed conflicts, 1946–2009. J Peace Res. 2010; 47: 501–509. 10.1177/0022343310376887

[2] Cloke K. Mediating Evil, War, and Terrorism: The Politics of Conflict In: Burgess G, Burgess H, editors. Beyond Intractability. Boulder, CO: Conflict Information Consortium, University of Colorado, Boulder; 2005 Available: http://www.beyondintractability.org/essay/mediating-evil. Accessed 8 September 2015.

[3] Barber BK, Schluterman JM. An overview of the empirical literature on adolescents and political violence In Barber BK, editor. Adolescents and war: How youth deal with political violence. New York, NY: Oxford University Press; 2009 pp. 35–61.

[4] Barber BK. Research on youth and political conflict: Where is the politics? Where are the youth? Child Dev Perspect. 2014; 8: 125–130. 10.1111/cdep.12074

[5] Pedersen D, Tremblay J, Errazuriz C, Gamarra J. The sequelae of political violence: assessing trauma, suffering and dislocation in the Peruvian highlands. Soc Sci Med. 2008; 67(2): 205–217. 10.1016/j.socscimed.2008.03.040 18423959

[6] Gilligan C. ‘Highly vulnerable’? Political violence and the social construction of traumatized children. J Peace Res. 2009; 46(1): 119–134. 10.1177/0022343308098407

[7] Neuner F. Assisting war-torn populations—should we prioritize reducing daily stressors to improve mental health? Comment on Miller and Rasmussen. Soc Sci Med. 2010; 71(8): 1381–1384; discussion 1385–1389. 10.1016/j.socscimed.2010.06.030 20674115

[8] Panter-Brick C, Eggerman M, Gonzalez V, Safdar S. Violence, suffering, and mental health in Afghanistan: a school-based survey. Lancet 2009; 374(9692): 807–816. 10.1016/S0140-6736(09)61080-1 19699514 PMC2748901

[9] Summerfield DA. Coping with the aftermath of trauma: NICE guidelines on post-traumatic stress disorder have fundamental flaw. BMJ 2005; 331(7507): 50; author reply: 50. 10.1136/bmj.331.7507.50-a PMC558579 15994701

[10] Batniji R. Searching for dignity. Lancet 2012; 380 (9840): 466–467. 22870512 10.1016/s0140-6736(12)61280-x

[11] Horwitz AV, Wakefield JC. The loss of sadness: How psychiatry transformed normal sorrow into depressive disorder. New York, NY: Oxford University Press; 2007. 10.1176/appi.ajp.2007.07081263 22688233

[12] Muldoon O, Lowe RD. Social identity, groups, and post-traumatic stress disorder. Political Psychol. 2012; 33: 259–273. 10.1111/pops.12709 PMC8247337 34219849

[13] Prilleltensky I. Wellness as fairness. Am J Commun Psychol. 2012; 49(1–2): 1–21. 10.1007/s10464-011-9448-8 21643926

[14] Barber BK, El Sarraj E, McNeely C, Daher M, Giacaman R, Arafat C, Barnes W, Abu Mallouh M. Feeling broken or destroyed: A mixed-methods study of contextualized suffering in the oPt. Lancet 2015; in press.

[15] Barber BK, Olsen JA. Positive and negative psychosocial functioning after political conflict: Examining adolescents of the first Palestinian Intifada In Barber BK, editor. Adolescents and war: How youth deal with political violence. New York, NY: Oxford University Press; 2009 pp. 207–237.

[16] B’Tselem. The Palestinian economy during the period of the Oslo Accords: 1994–2000. 2011 Jan 1 [cited 8 September 2015]. In: Economic & Social Rights [Internet]. Jerusalem: B'Tselem, The Israeli Information Center for Human Rights in the Occupied Territories. Available: http://www.btselem.org/freedom_of_movement/economy_1994_2000

[17] United Nations Conference on Trade and Development (UNCTAD). The Palestinian economy: Macroeconomic and trade policymaking under occupation. New York, NY: United Nations; 2012 Available: http://unctad.org/en/PublicationsLibrary/gdsapp2011d1_en.pdf. Accessed 8 September 2015.

[18] McNeely C, Barber BK, Giacaman R, Arafat C, Daher M, El Sarraj E, Abu-Mallouh M, Belli R. Political imprisonment and adult functioning: A life event history analysis of Palestinians. J Trauma Stress 2015; 28(3): 223–231. 10.1002/jts.22015 26062134

[19] Abu-Zahra N, Kay A. Unfree in Palestine: Registration, Documentation and Movement Restriction. London: Pluto Press; 2012.

[20] Assaf N. West Bank and Gaza—Investment climate assessment: fragmentation and uncertainty. Washington, DC: World Bank Group; 2014 Available: http://documents.worldbank.org/curated/en/2014/01/20189765/west-bank-gaza-investment-climate-assessment-fragmentation-uncertainty. Accessed 4 September 2015.

[21] United Nations Commission on Trade and Development (UNCTAD). Report on UNCTAD assistance to the Palestinian people: Developments in the economy of the Occupied Palestinian Territory. Geneva: United Nations; 2015 Available: http://unctad.org/meetings/en/SessionalDocuments/tdb62d3_en.pdf. Accessed 4 September 2015.

[22] Rytter MJH, Kjældgaard AL, Brønnum-Hansen H, Helweg-Larsen K. Effects of armed conflict on access to emergency health care in Palestinian West Bank: Systematic collection of data in emergency departments. BMJ 2006; 332:1122–4. 10.1136/bmj.38793.695081.AE 16585049 PMC1459547

[23] United Nations. Gaza 2020: A livable place? A report by the United Nations Country Team in the occupied Palestinian territory. Jerusalem: Office of the United Nations Special Coordinator for the Middle East Peace Process (UNSCO); 2012 Available: http://www.unrwa.org/userfiles/file/publications/gaza/Gaza%20in%202020.pdf. Accessed 4 September 2015.

[24] Miller KE, Omidian P, Quraishy AS, Quraishy N, Nasiry MN, Nasiry S, et al The Afghan symptom checklist: a culturally grounded approach to mental health assessment in a conflict zone. Am J Orthopsychiatr. 2006; 76(4): 423–433. 10.1037/0002-9432.76.4.423 17209710

[25] Eggerman M, Panter-Brick C. Suffering, hope, and entrapment: resilience and cultural values in Afghanistan. Soc Sci Med. 2010; 71(1):71–83. 10.1016/j.socscimed.2010.03.023 20452111 PMC3125115

[26] Tol WA, Komproe IH, Jordans MJD, Susanty D, De Jong JTVM. Developing a function impairment measure for children affected by political violence: A mixed methods approach in Indonesia. Int. J Qual Health Care 2011; 23(4): 375–383. 10.1093/intqhc/mzr032 et al 2011 21676960

[27] Betancourt TS. A qualitative study of mental health problems among children displaced by war in Northern Uganda. Transcult Psychiatry 2009; 46: 238–256. 10.1177/1363461509105815 19541749 PMC2775515

[28] Neuner F, Pfeiffer A, Schauer-Kaiser E, Odenwald M, Elbert T, Ertl V. Haunted by ghosts: Prevalence, predictors and outcomes of spirit possession experiences among former child soldiers and war-affected civilians in Northern Uganda. Soc Sci Med. 2012; 75: 548–554. 10.1016/j.socscimed.2012.03.028 22580073

[29] Betancourt TS, Williams TP, Kellner SE, Gebre-Medhin J, Hann K, Kayiteshonga Y. Interrelatedness of child health, protection and well-being: An application of the SAFE model in Rwanda. Soc Sci Med. 2012; 74(10): 1504–1511. 10.1016/j.socscimed.2012.01.030 22459187

[30] Rasmussen A, Keatley E, Joscelyne A. Posttraumatic stress in emergency settings outside North America and Europe: A review of the emic literatures. Soc Sci Med. 2014; 109: 44–54. 10.1016/j.socscimed.2014.03.015 24698712 PMC4070307

[31] World Health Organization. International Classification of Functioning. Geneva, Switzerland: World Health Organization; 2001.

[32] Barber BK, Spellings C, McNeely C, Page PD, Giacaman R, Arafat C, Daher M, El Sarraj E, Abu Mallouh M. Politics drives human functioning, dignity, and quality of life. Soc Sci Med. 2014; 122: 90–102. 10.1016/j.socscimed.2014.09.055 25441321

[33] Corbin J, Strauss A. Basics of qualitative research: Grounded theory procedures and techniques. 3rd ed Thousand Oaks, CA: Sage Publications; 2008.

[34] McNeely C, Barber BK, Spellings C, Giacaman R, Arafat C, Daher M, et al Human insecurity, chronic economic constraints and health in the occupied Palestinian territory. Glob Public Health 2014; 9(5): 495–515. 10.1080/17441692.2014.903427 24766078

[35] Kroenke K, Spitzer RL. The PHQ-9: A new depression diagnostic and severity measure. Psychiatr Ann. 2002; 32: 509–515.

[36] Foa EB, Riggs DS, Dancu CV, Rothbaum B. Reliability and validity of a brief instrument for assessing posttraumatic stress-disorder. J. Trauma. Stress 1993; 6: 459–473. 10.1002/jts.2490060405

[37] Fernando GA. Assessing mental health and psychosocial status in communities exposed to traumatic events: Sri Lanka as an example. Am J Orthopsychiat 2008; 78:229–239. 10.1037/a0013940 18954186

[38] Rasmussen A, Katoni B, Keller AS, Wilkinson J. Posttraumatic idioms of distress among Darfur refugees: *Hozun* and *Majnun*. Transcult Psychiatry 2011; 48:392–415. 10.1177/1363461511409283 21911508

[39] Barber BK, Billen R, Youniss J. The psychology of political control: Measuring insecurity and feeling broken in post-revolution Egypt. Paper to be presented at the annual meeting of the International Society for Political Psychology, July 2015. San Diego, CA.

[40] Tessler MA. A history of the Israeli-Palestinian conflict. 2nd ed Bloomington, IN: Indiana University Press; 2009.

[41] Shachar N. The Gaza Strip: Its history and politics: From the pharaohs to the Israeli invasion of 2009. Portland, OR: Sussex Academic Press; 2010.

[42] United Nations. Gaza in 2020: A livable place? A report by the United Nations Country Team in the occupied Palestinian territory. 2012. Jerusalem, occupited Palestinian territory: Office of the United Nations Special Coordinator for the Middle Ease Peace Process. Available: http://www.unrwa.org/userfiles/file/publications/gaza/Gaza%20in%202020.pdf.

[43] Dabbagh NT. Narrative expressions of despair under occupation. Anthropol Med. 2004; 11(2): 201–220. 10.1080/13648470410001678686 26868202

[44] Dowty A. Despair is not enough: Violence, attitudinal change, and 'ripeness' in the Israeli-Palestinian conflict. Coop Confl. 2006; 41(1): 5–29. 10.1177/0010836706060930

[45] Hanafi S, Long T. Governance, governmentalities, and the state of exception in the Palestinian refugee camps of Lebanon. J of Refug Stud. 2010; 23(2): 134–159. 10.1093/jrs/feq014

[46] Peteet J. Landscape of hope and despair: Palestinian refugee camps. Philadelphia, PA: University of Pennsylvania Press; 2005.

[47] Roy S. The political economy of despair: changing political and economic realities in the Gaza Strip. J of Palest Stud. 1991; 20(3): 58–69.

[48] Berger J. Dispatches: Undefeated despair. Race Cl. 2006; 48(1): 23–41. 10.1177/0306396806066645

[49] Hass A. Drinking the sea at Gaza. New York: Metropolitan Books; 1996.

[50] El Sarraj E. In search of dignity. Lancet 2012; 380: 1386 23084455 10.1016/S0140-6736(12)61802-9

[51] Ehlers A, Maercker A, Boos A. Posttraumatic stress disorder following political imprisonment: The role of mental defeat, alienation, and perceived permanent change. J Abnorm Psychol. 2000; 109: 45–55. 10.1037//0021-843X.109.1.45 10740935

[52] Bolger E. Grounded theory analysis of emotional pain. Psychother Res. 1999; 9(3): 342–362. 10.1080/10503309912331332801

[53] El Sarraj E. Defiant, helpless and demoralized. Palest-Isr J. 2003; 10(4). Available: http://www.pij.org/details.php?id=62

[54] Jacobsen JC, Maytal G, Stern TA. Demoralization in medical practice. J Clin Psychiatry 2007; 9(2): 139–143. 10.4088/PCC.v09n0208 PMC1896303 17607336

[55] Maslach C, Schaufeli WB, Leiter MP. Job Burnout. Annu Rev Psychol. 2001; 52: 397–422. 11148311 10.1146/annurev.psych.52.1.397

[56] Schaufeli WB, Leiter MP, Maslach C. Burnout: 35 Years of Research and Practice. Career Dev Int. 2008; 14(3): 204–220. 10.1108/13620430910966406

[57] Almedom AM, Summerfield D. Mental well-being in settings of ‘complex emergency’: An overview. J Biosoc Sci. 2004; 36: 381–388. 10.1017/S0021932004006832 15293381

[58] Giacaman R, Abu-Rmeileh NME, Husseini A, Saab H, Boyce W. Humiliation: The invisible trauma of war for Palestinian youth. Public Health 2007; 121: 563–571. 10.1016/j.puhe.2006.10.021 17568641

[59] Giacaman R, Rabaia Y, Nguyen-Gillham V, Batniji R, Punamaki RL, Summerfield D. Mental health, social distress and political oppression: The case of the occupied Palestinian territory. Glob Public Health 2011; 6: 547–559. 10.1080/17441692.2010.528443 21108104

[60] Giacaman R, Johnson P. Our life in prison: The triple captivity of wives and mothers of Palestinian political prisoners. J Middle East Women’s Stud. 2013; 9: 54–80.

[61] Kleinman A, Das V, & Lock MM. Social suffering. Berkeley: University of California Press; 1997.

[62] Farmer P. An anthropology of structural violence. Curr. Anthropol. 2004; 45(3): 305–325.

[63] Barber BK, McNeely C, Olsen JA, Spellings C, Belli RF. Effect of chronic exposure to humiliation on wellbeing in the occupied Palestinian territory: an event-history analysis. Lancet 2013; 382, Supplement 4(0): S7 10.1016/S0140-6736(13)62579-9

[64] Barber BK., McNeely CM, Olsen JA, Belli RF, Doty SB. Long-term exposure to political violence: The particular injury of persistent humiliation. Soc Sci Med. 2016; 156: 154–166. 10.1016/j.socscimed.2016.03.011 27039017

